# Presence of an interferon signature in individuals who are anti-nuclear antibody positive lacking a systemic autoimmune rheumatic disease diagnosis

**DOI:** 10.1186/s13075-017-1243-y

**Published:** 2017-02-28

**Authors:** Joan Wither, Sindhu R. Johnson, Tony Liu, Babak Noamani, Dennisse Bonilla, Larissa Lisnevskaia, Earl Silverman, Arthur Bookman, Carolina Landolt-Marticorena

**Affiliations:** 1 Krembil Research Institute, University Health Network, Toronto, ON Canada; 2 Division of Rheumatology, University Health Network, Toronto, ON Canada; 3 Department of Medicine, University of Toronto, Toronto, ON Canada; 4 Department of Immunology, University of Toronto, Toronto, ON Canada; 5 Toronto Western Hospital, 1E-420, 399 Bathurst Street, Toronto, ON M5T 2S8 Canada; 6 Division of Rheumatology, Mount Sinai Hospital, Toronto, ON Canada; 7 Institute of Health Policy, Management and Evaluation, University of Toronto, Toronto, ON Canada; 8 Lakeridge Heath Services, Oshawa, ON Canada; 9 Division of Rheumatology, Hospital for Sick Children, Toronto, ON Canada; 10 Department of Pediatrics, University of Toronto, Toronto, ON Canada

**Keywords:** Systemic autoimmune rheumatic disease, Anti-nuclear antibodies, Pre-clinical, Interferon

## Abstract

**Background:**

Elevated levels of type I interferons (IFNs) are a characteristic feature of the systemic autoimmune rheumatic diseases (SARDs) and are thought to play an important pathogenic role. However, it is unknown whether these elevations are seen in anti-nuclear antibody–positive (ANA^+^) individuals who lack sufficient criteria for a SARD diagnosis. We examined IFN-induced gene expression in asymptomatic ANA^+^ individuals and patients with undifferentiated connective tissue disease (UCTD) to address this question.

**Methods:**

Healthy ANA^−^ control subjects and ANA^+^ titre (≥1:160 by immunofluorescence) participants meeting no criteria, meeting at least one criterion (UCTD) or meeting SARD classification criteria were recruited. Whole peripheral blood IFN-induced and *BAFF* gene expression were quantified using NanoString technology. The normalized levels of five IFN-induced genes were summed to produce an IFN5 score.

**Results:**

The mean IFN5 scores were increased in all ANA^+^ participant subsets as compared with healthy control subjects. We found that 36.8% of asymptomatic ANA^+^ and 50% of UCTD participants had IFN5 scores >2 SD above the mean for healthy control subjects. In all ANA^+^ subsets, the IFN5 score correlated with the presence of anti-Ro/La antibodies. In the asymptomatic ANA^+^ subset, this score also correlated with the ANA titre, whereas in the other ANA^+^ subsets, it correlated with the number of different ANA specificities. Development of new SARD criteria was seen in individuals with normal and high IFN5 scores.

**Conclusions:**

An IFN signature is seen in a significant proportion of ANA^+^ individuals and appears to be associated with ANA titre and type of autoantibodies, rather than with the presence or development of clinical SARD symptoms.

## Background

The systemic autoimmune rheumatic diseases (SARDs) that are frequently associated with a positive anti-nuclear antibody (ANA) test result include systemic lupus erythematosus (SLE), Sjögren’s syndrome (SS), systemic sclerosis (SSc), dermatomyositis (DM) and mixed connective tissue disease (MCTD). Although each of these conditions constitutes a distinct clinical syndrome, they all share a similar etiopathogenesis based upon their overlapping clinical features, co-segregation within families and shared production of ANAs [1–4]. One of the characteristic features of these conditions is a prolonged pre-clinical phase during which autoantibodies can be detected in the absence of clinical disease. In SLE, autoantibodies can be detected up to 9 years before development of clinical symptoms [5], and similar observations have been made in SSc and SS [6, 7]. Because the onset of clinical symptoms in these conditions can be associated with significant morbidity, and occasionally mortality, there is tremendous interest in identifying and treating patients with SARD earlier in their disease course [8, 9]. However, a major impediment to this approach is the observation that ANAs can also be seen in healthy individuals, particularly women, most of whom will not progress to SARD [10]. Thus, there is a need for additional biomarkers that can accurately predict individuals with a high likelihood of progression to SARD.

A number of immunologic changes have been observed at the onset of SARD. One of the most distinctive immunologic abnormalities is high levels of interferon (IFN)-induced gene expression, the so-called IFN signature [11–16]. Although initially described in patients with SLE, this signature was subsequently reported for all SARDs, with multiple lines of evidence suggesting that elevated levels of type I IFNs play an important pathogenic role in these conditions. Types II and III IFNs can also induce this signature, and there is emerging evidence that they may also play a role in disease pathogenesis [17]. Currently, it is not known whether individuals who are ANA^+^ who either lack symptoms or have insufficient symptoms to diagnose a SARD have an IFN signature. In this study, we assessed IFN-induced gene expression in these individuals and examined the serologic and clinical correlates.

## Methods

### Subjects and data collection

ANA^+^ individuals (titre ≥1:160 by immunofluorescence) were recruited at the Toronto Western and Mount Sinai hospitals, where they were assessed by one of the participating rheumatologists. The majority of patients were referred to these clinics because they had a recent positive ANA test result at an outside laboratory with or without rheumatologic symptoms. All clinical data were obtained through the use of a standardized questionnaire and a data retrieval form that elicited all of the clinical symptoms and signs required for disease classification. On the basis of their clinical and laboratory findings at their initial assessments, participants were stratified into three groups: (1) asymptomatic ANA^+^ individuals lacking any clinical symptoms of SARD, (2) patients with undifferentiated connective tissue disease (UCTD) with at least one clinical symptom of SARD and (3) patients with early SARD meeting classification criteria (1997 American College of Rheumatology [ACR] criteria for SLE [18], 2013 ACR-European League Against Rheumatism [EULAR] criteria for SSc [19] or the revised American-European criteria for SS [20]) and who had received their diagnosis within the previous 2 years. To avoid inclusion of patients with long-standing disease who had only recently been diagnosed with SS on the basis of the established criteria, only patients whose symptoms had begun within the previous 5 years were included in the study. All patients were steroid-naïve and off disease-modifying anti-rheumatic drugs (DMARDs), with the exception of anti-malarials. Age- and sex-matched healthy control subjects (HC) were recruited from among hospital/laboratory personnel and were ANA- and specific anti-nuclear autoantibody- negative. The study was approved by the research ethics boards of both recruiting hospitals, and all participants signed informed consent forms.

### RNA isolation and performance of NanoString

Total RNA was isolated from whole peripheral blood archived in Tempus tubes using a Tempus Spin RNA Isolation Kit (Applied Biosystems, Foster City, CA, USA) following the manufacturer’s instructions. Gene expression was quantified using NanoString technology with 100 ng of RNA in a custom array (NanoString Technologies, Seattle, WA, USA) at the Farncombe Metagenomics Facility (McMaster University, Hamilton, ON, Canada). The expression levels of five IFN-induced genes (*EPSTI1*, *IFI44L*, *LY6E*, *OAS3*, and *RSAD2)* that were previously reported to be induced by IFN-α and ubiquitously expressed in multiple cell types were measured and summed to generate an IFN5 score, which was used as the primary measure of an IFN signature. Expression of two IFN-induced genes that are reported to indicate stronger IFN-induced gene induction (*EIF2AK2* and *PLSCR1*) and *BAFF* were also assessed. Raw expression levels of all genes were normalized to expression of five housekeeping genes (*FPGS*, *HPRT1*, *GAPDH*, *PPIB*, and *TBP*) using nSolver software (NanoString Technologies).

### Measurement of autoantibodies

All participants had their ANA quantified by indirect immunofluorescence with serum obtained at the time of recruitment through the University Health Network laboratory, which uses HEp-2 cells as a substrate. Only individuals with an ANA titre ≥1:160 at this determination were included in the ANA^+^ study cohorts. The serum levels of ten specific autoantibodies (anti-double-stranded DNA [anti-dsDNA], anti-chromatin, anti-Ro, anti-La, anti-Smith [anti-Sm], anti-Sm/RNP, anti-ribonuclear protein [anti-RNP], anti-Jo-1, anti-Scl-70 and anti-centromere) were assayed by the BioPlex® 2200 ANA Screening System (Bio-Rad Laboratories, Hercules, CA, USA) in the hospital laboratory using the manufacturer’s cut-offs. Healthy control subjects with an ANA titre ≥1:160 were re-classified into the asymptomatic ANA^+^ group (25.7% of female and 0% of male healthy control subjects recruited) and those with a positive ANA at a titre <1:160 or any specific autoantibodies (8.6% of female and 0% of male healthy control subjects recruited) were excluded from the study.

### Measurement of serum IFN-α

Serum archived at the time of recruitment was stored at −80 °C and thawed immediately prior to testing. Serum levels of IFN-α were measured in duplicate using the VeriKine-HS Human Interferon Alpha All Subtype ELISA Kit (PBL Assay Science, Piscataway, NJ, USA). The lower limit of detection of this kit is 1.95 pg/ml.

### Statistical analysis

For comparisons of differences between three or more groups, a Kruskal-Wallis test was used, followed by Dunn’s post-test for multiple comparisons. When two groups were compared, a Mann-Whiney *U* test was performed for continuous variables, and a χ^2^ or Fisher’s exact test was used for discrete variables. The strength of association between variables was determined using Spearman’s correlation coefficient. All statistical analyses were performed using Prism 6 software (GraphPad Software, La Jolla, CA, USA).

## Results

### A significant number of ANA^+^ participants without a SARD diagnosis have elevated levels of IFN-induced gene expression

Participant demographics are shown in Table 1. There were no significant differences in the sex, age or proportion of participants taking anti-malarials between groups. Several of the asymptomatic ANA^+^ individuals were taking anti-malarials for symptoms (fatigue, arthralgia/myalgia) that could not be definitively attributed to SARD. Although participants with early SARD could be within 2 years of receiving their diagnosis, owing to the requirement for no prednisone or DMARD treatment, the majority of patients were recruited at initial presentation, with the exception of patients with SS (≤5 years from symptom onset).

**Table 1:** Study participant characteristics

	Healthy control subjects (*n* = 20)	ANA^+^ no symptoms (*n* = 38)	UCTD (*n* = 28)	SARD (*n* = 58)	SSc (*n* = 26)	SLE (*n* = 6)	SS (*n* = 23)	DM/MCTD (*n* = 3)
Female sex, *n* (%)	18 (90)	37 (97.4)	27 (96.4)	54 (93.1)	24 (92.3)	6 (100)	21 (91.3)	3 (100)
Age, years, mean ± SD	41 ± 12.4	44.1 ± 14.3	47.5 ± 15.4	51.5 ± 14.4	52.8 ± 14.7	39 ± 12.3	54.8 ± 12.7	39.3 ± 6.6
Anti-malarials, *n* (%)	0 (0)	5 (13.2)	4 (14.3)	7 (12.1)	2 (7.7)	2 (33.3)	2 (8.7)	1 (33.3)
Ethnicity, *n* (%)								
Caucasian	9 (45)	23 (60.5)	20 (71.4)	41 (70.7)	18 (69.2)	4 (66.7)	17 (73.9)	2 (66.7)
African	1 (5)	4 (10.5)	3 (10.7)	0 (0)	0 (0)	0 (0)	0 (0)	0 (0)
Asian	1 (5)	1 (2.6)	3 (10.7)	3 (5.2)	1 (3.8)	0 (0)	2 (8.7)	0 (0)
Southeast Asian	3 (15)	5 (13.2)	0 (0)	7 (12.1)	3 (11.5)	2 (33.3)	2 (8.7)	0 (0)
Filipino	4 (20)	1 (2.6)	1 (3.6)	4 (6.9)	3 (11.5)	0 (0)	0 (0)	1 (33.3)
Hispanic	1 (5)	1 (2.6)	1 (3.6)	0 (0)	0 (0)	0 (0)	0 (0)	0 (0)
Other	1 (5)	3 (7.9)	0 (0)	3 (5.2)	1 (3.8)	0 (0)	2 (8.7)	0 (0)
Specific antibodies, *n* (%)								
dsDNA	0 (0)	2 (5.3)	4 (14.3)	9 (15.5)	3 (11.5)	2 (33.3)	3 (13.0)	1 (33.3)
Ro	0 (0)	7 (18.4)	8 (28.6)	30 (51.7)	4 (15.4)	3 (50)	23 (100)	0 (0)
La	0 (0)	2 (5.3)	4^a^ (14.3)	18 (31.0)	0 (0)	1 (16.7)	17 (73.9)	0 (0)
Sm	0 (0)	0 (0)	2 (7.1)	3 (5.2)	0 (0)	2 (33.3)	0 (0)	1 (33.3)
Sm/RNP	0 (0)	0 (0)	5 (17.9)	6 (10.3)	2 (7.7)	2 (33.3)	0 (0)	2 (66.7)
RNP	0 (0)	4 (10.5)	4 (14.3)	8 (13.8)	2 (7.7)	3 (50)	1 (4.3)	2 (66.7)
Scl-70	0 (0)	0 (0)	2 (7.1)	10 (17.2)	7 (26.9)	1 (16.7)	2 (8.7)	0 (0)
Jo-1	0 (0)	0 (0)	0 (0)	0 (0)	0 (0)	0 (0)	0 (0)	0 (0)
Centromere	0 (0)	1 (2.6)	1 (3.6)	17 (29.3)	14 (53.8)	1 (16.7)	1 (4.3)	1 (33.3)
Chromatin	0 (0)	2 (5.3)	4 (14.3)	6 (10.3)	1 (3.8)	3 (50)	0 (0)	2 (66.7)

*Abbreviations*: *ANA* Anti-nuclear antibody, *UCTD* Undifferentiated connective tissue disease, *SARD* Systemic autoimmune rheumatic disease, *SSc* Systemic sclerosis, *SS* Sjögren’s syndrome, *SLE* Systemic lupus erythematosus, *DM* Dermatomyositis, *MCTD* Mixed connective tissue disease, *dsDNA* Double-stranded DNA, *Sm* Smith, *RNP* Ribonuclear protein

^a^All patients that were anti-La antibody positive were anti-Ro antibody positive, except for 1 patient with UCTD

**Fig. 1:**
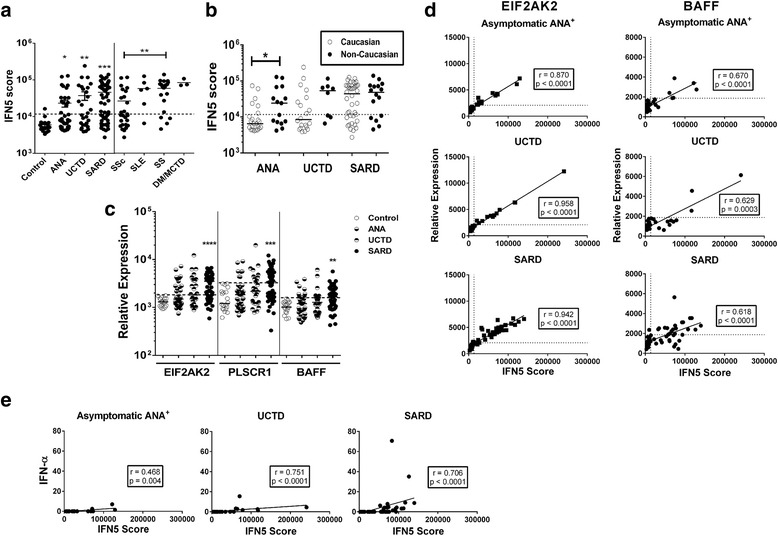
Levels of interferon (IFN)-induced gene expression in the participant subsets, stratified by diagnosis. **a** IFN-induced gene expression was quantified in whole peripheral blood RNA using NanoString technology and the normalized levels of five ubiquitously expressed IFN-induced genes summed to produce an IFN5 score. Results to the *left* of the figure are shown for healthy control subjects (Control), individuals who were asymptomatic anti-nuclear antibody–positive (ANA^+^), and patients who had undifferentiated connective tissue disease (UCTD) and early systemic autoimmune rheumatic disease (SARD). Significant differences from healthy control subjects are indicated as * *p* < 0.05, ** *p* < 0.01 and *** *p* < 0.001. Results to the *right* of the figure show the IFN5 scores for the different early SARD patient subsets with the significant differences between groups indicated. **b** IFN5 scores for different ANA^+^ subject subsets, stratified by ethic group. **c** Expression levels of two IFN-β-induced genes (*EIF2AK2* and *PLSCR1*) and *BAFF* in whole peripheral blood. Significant differences from healthy control subjects are indicated. **d** Correlation between *EIF2AK2* and *BAFF* levels and IFN5 score for the different ANA^+^ subsets. *Solid lines* indicate linear regression curves. For all panels, *dashed lines* represent 2 SD above the mean for healthy control subjects. **e** Correlation between serum IFN-α levels as measured by enzyme-linked immunosorbent assay and IFN5 scores for the different ANA^+^ subsets. *Solid lines* indicate linear regression curves. *SSc* Systemic sclerosis, *SS* Sjögren’s syndrome, *SLE* Systemic lupus erythematosus, *DM* Dermatomyositis, *MCTD* Mixed connective tissue disease

IFN-induced gene expression was first assessed using the IFN5 score, the sum of normalized gene expression for five genes that are increased in multiple SLE patient peripheral blood mononuclear cell subsets [21]. Asymptomatic and UCTD non-SARD ANA^+^ participants had elevated levels of IFN-induced gene expression as compared with ANA^−^ HC (Fig. 1a). Although the mean IFN5 score was lower in non-SARD ANA^+^ participants than in patients with SARD, a number of individuals in both non-SARD groups had levels comparable to those seen in SARD. Overall, 36.8% of asymptomatic ANA^+^ subjects and 50% of patients with UCTD had IFN5 scores that were >2 SD above the mean for HC. Treatment with anti-malarials did not appear to be associated with any consistent differences in IFN5 levels. One of five ANA^+^ individuals without SARD clinical diagnostic criteria and two of four patients with UCTD taking anti-malarials had high IFN5 levels in the same range as those patients who were not on treatment. Of note, high IFN5 scores were seen not only in those patients referred to a rheumatologist but also in individuals recruited as HC who were subsequently reclassified as ANA^+^ following ANA testing. In early SARD, 65.5% of patients had elevated IFN5 scores (SSc 42.3%, SS 82.6%, SLE 80.3%, MCTD/DM 100%), with patients with SSc having lower IFN5 scores than those in the other SARD groups.

There was no association between age or sex and IFN5 levels in any of the ANA^+^ groups. With regard to ethnicity, as shown in Table 1, the majority of study participants were Caucasian. Previous work has indicated that high levels of IFN are more likely to be driven by autoantibodies in individuals of non-European ancestry who have SLE [22, 23]. Therefore, to assess whether there was an association between ethnicity and IFN-induced gene expression, the IFN5 score was compared between Caucasian subjects and those of all other ethnicities. As shown in Fig. 1b, within the subset of ANA^+^ individuals lacking SARD classification criteria, the mean IFN5 score was higher in non-Caucasian subjects than in Caucasian subjects. A similar non-significant trend was seen for patients with UCTD, but this was lost in patients with early SARD. Notably, not only African but also Asian and Southeast Asian ethnicities were enriched in the non-SARD subgroup of individuals with high IFN5 scores.

It has been suggested that the IFN-induced genes that are typically included in composite IFN scores (including those in the IFN5 score) are driven predominantly by IFN-α and may reflect a weaker IFN signature than genes contained in clusters that may be driven by other IFNs, such as IFN-β [17]. Therefore, to determine whether the IFN signature seen in ANA^+^ individuals that lack a SARD diagnosis differs from that seen in early SARD, expression levels of two genes that are contained within the cluster of genes that have been reported to be induced by IFN-β and elevated in patients with SLE who have higher levels of IFN-induced gene expression [17], *EIF2AK2* and *PLSCR1*, were examined. In addition, to explore whether the levels of IFN in patients without SARD were sufficient to induce other cytokines/chemokines in vivo, peripheral blood *BAFF* expression was assessed. The levels of all three genes were significantly elevated in patients with early SARD (Fig. 1c) and showed a moderate (*BAFF*) to strong (*EIF2AK2* and *PLSCR1*) correlation with the IFN5 score (Fig. 1d and data not shown). Although the mean levels of these genes were not significantly increased in asymptomatic ANA^+^ individuals or patients with UCTD (Fig. 1c), the same strong correlation between their expression levels and IFN5 scores was seen (Fig. 1d), with a significant proportion of both subsets of subjects having levels >2 SD above the mean for HC (percent elevated *EIF2AK2*, *PLSCR1* and *BAFF*: asymptomatic ANA^+^ 34.2%, 18.4%, and 28.6%, respectively; UCTD 50%, 35.7% and 25%, respectively). These findings indicate strong induction of IFN-induced genes in a subset of non-SARD ANA^+^ individuals and suggest that the IFN-induced gene expression observed in a significant proportion of ANA^+^ individuals without SARD does not differ quantitatively or qualitatively from that observed in patients with early SARD or that previously reported for patients with SLE [17].

Previous data suggest that there is a moderate correlation between serum IFN activity and IFN-induced gene expression in SLE. Therefore, to determine whether the IFN-induced gene expression observed in individuals with early SARD and ANA^+^ individuals without SARD is similarly correlated with serum IFN levels, we measured serum IFN-α using a high-sensitivity enzyme-linked immunosorbent assay that detects all IFN-α subtypes. As shown in Fig. 1e, there was a moderate correlation between serum IFN-α levels and IFN5 scores in the early SARD and both non-SARD ANA^+^ groups. Notably, despite the use of a high-sensitivity assay, serum levels of IFN-α were below the limit of detection for all samples from individuals with IFN5 scores <50,000, regardless of diagnosis. There was no difference between the different ANA^+^ groups in the proportion of individuals with scores greater than this who had detectable levels of serum IFN-α, indicating that ANA^+^ individuals without SARD who had high IFN5 scores are just as likely to have elevated levels of serum IFN-α as those with early SARD.

### In ANA^+^ participants lacking a SARD diagnosis, the levels of IFN-induced gene expression correlate with serologic findings

**Fig. 2:**
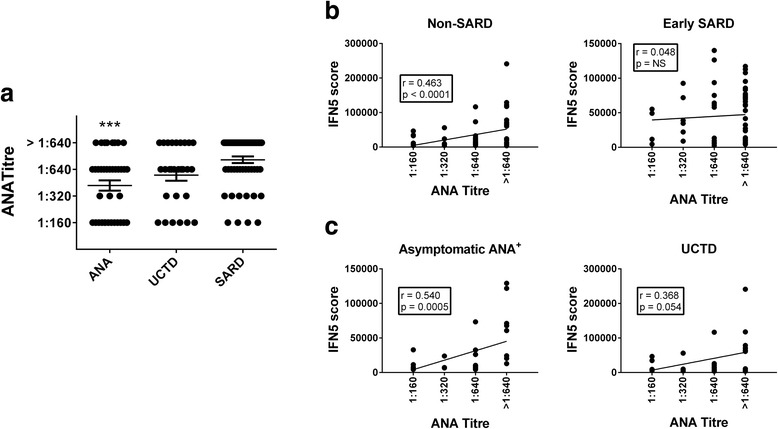
Association between anti-nuclear antibody (ANA) titre and interferon (IFN)-induced gene expression. **a** ANA titres in asymptomatic ANA^+^ individuals, patients with undifferentiated connective tissue disease (UCTD) and patients with early systemic autoimmune rheumatic disease (SARD). Significant differences from patients with early SARD are indicated as *** *p* < 0.001. **b** Association between IFN5 score and ANA titre in participants with non-SARD (asymptomatic ANA^+^ and UCTD) and those with early SARD. **c** Association between IFN5 scores and ANA titre for asymptomatic ANA^+^ participants and patients with UCTD. Significant associations are indicated

To determine whether differences in the levels of IFN-induced gene expression were due to variations in the ANA titre and the type and number of specific autoantibodies, the association between the IFN5 score and these serologic variations was assessed. Figure 2a shows the ANA titres in each ANA^+^ participant subset. The mean ANA titre was significantly lower in asymptomatic ANA^+^ individuals than in those with early SARD. There was no association between ANA titre and ethnicity in any of the groups examined. When all ANA^+^ individuals were examined, there was a modest correlation between ANA titre and IFN5 levels (*r* = 0.38, *p* < 0.0001). This appeared to be driven by the non-SARD ANA^+^ subset, and particularly the asymptomatic group (Fig. 2b and c). In the asymptomatic ANA^+^ group, this correlation remained present when just the subset of individuals with no specific autoantibodies was examined (*r* = 0.609, *p* = 0.0021).

**Fig. 3:**
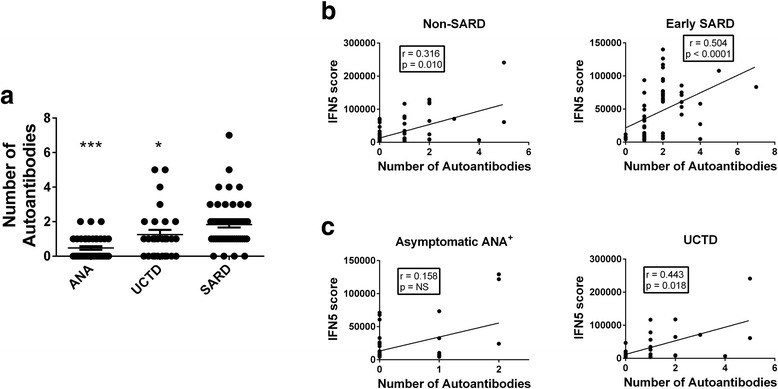
Association between the number of different anti-nuclear antibody (ANA) specificities detected and interferon (IFN)-induced gene expression. **a** The number of different ANA specificities detected using the BioPlex® 2200 system in asymptomatic ANA^+^ individuals, participants with undifferentiated connective tissue disease (UCTD), and participants with early systemic autoimmune rheumatic disease (SARD). Significant differences from patients with early SARD are indicated as * *p* < 0.05, *** *p* < 0.001. Association between IFN5 score and the number of autoantibodies detected in (**b**) non-SARD (asymptomatic ANA^+^ and UCTD) and early SARD participants and in (**c**) asymptomatic ANA^+^ individuals and participants with UCTD. Significant associations are indicated

Fifty percent of non-SARD participants had at least one specific autoantibody, with the type and prevalence of specific autoantibodies varying somewhat between the different ANA^+^ groups (Table 1). Within each patient group, the number of different autoantibody specificities detected for asymptomatic ANA^+^ individuals or patients with UCTD was significantly lower than that observed in patients with early SARD (Fig. 3a), and patients with UCTD had significantly more autoantibody specificities than asymptomatic ANA^+^ individuals (*p* = 0.016). There was no association between the number or type of autoantibody specificities and ethnicity. Although there was a trend to a higher mean IFN5 score in the subset of participants without SARD who had at least one specific autoantibody as compared with none (42,170 ± 53,422 vs. 16,421 ± 18,732, *p* = 0.088), elevated IFN5 scores were still seen in 33% of individuals with no specific autoantibodies. This proportion did not differ significantly from the proportion of individuals with at least one specific autoantibody who had high IFN5 scores (48.5%). Nevertheless, in both individuals without SARD and those with early SARD, there was a moderate correlation between the number of different autoantibody specificities and the IFN5 score (Fig. 3b), and this was also seen in the UCTD subset (Fig. 3c). Similar findings were observed for the association between the number of different autoantibody specificities and serum IFN-α levels in these groups (early SARD: *r* = 0.462, *p* = 0.0011; non-SARD: *r* = 0.539, *p* < 0.0001; UCTD: *r* = 0.668, *p* = 0.0002).

**Fig. 4:**
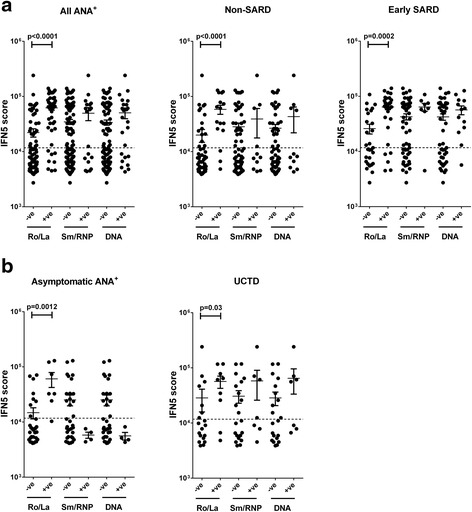
Correlation between interferon (IFN)-induced gene expression and the presence of specific anti-nuclear antibodies (ANA). The levels of specific ANA were measured using the BioPlex® 2200 ANA screening system, and participants were stratified on the basis of presence or absence of anti-Ro and/or anti-La (Ro/La), anti-Smith (anti-Sm) and/or anti-Sm/ribonuclear protein (RNP) and/or anti-RNP (Sm/RNP), or anti-double-stranded (anti-dsDNA) and/or anti-chromatin (DNA). Correlation between each cluster of autoantibodies and the IFN5 score for (**a**) all ANA^+^ participants and the non-systemic autoimmune rheumatic disease (SARD) and early SARD subsets and (**b**) asymptomatic ANA^+^ individuals and participants with undifferentiated connective tissue disease (UCTD). Significant correlations are indicated. *Dashed lines* in each figure represent 2 SD above the mean for healthy control subjects

To investigate whether particular types of autoantibodies are associated with elevated levels of IFN-induced gene expression, autoantibody profiles were clustered into three groups: Ro and/or La, RNP and/or Sm/RNP and/or Sm, and chromatin and/or dsDNA. Only the presence of anti-Ro and/or anti-La antibodies demonstrated a significant association with IFN5 levels, and this was seen for all ANA^+^ groups (Fig. 4). In Ro^+^ individuals, there was no correlation between the levels of anti-Ro antibodies and IFN5 levels, and the IFN5 levels were not significantly different between participants with and without anti-La antibodies (data not shown). Consistent with the association between anti-Ro and/or anti-La antibodies and high IFN5 levels, the majority (18 of 26) of individuals without SARD who had elevated IFN5 levels had a speckled pattern of immunofluorescence in their ANA test.

Although the IFN5 levels in asymptomatic ANA^+^ individuals that were anti-RNP and/or anti-Sm/RNP and/or anti-Sm positive, or anti-chromatin and/or anti-dsDNA positive were lower than those observed for similarly positive patients within the UCTD and early SARD groups, the number of different autoantibody specificities associated with these autoantibodies differed markedly between these groups. All of the individuals in the asymptomatic ANA^+^ group had only one specific autoantibody (either RNP, dsDNA or chromatin), whereas the number of different autoantibody specificities ranged from one to five for UCTD (mean 2.7 for Sm/RNP and 3.1 for DNA) and from one to seven for early SARD (mean 4 for Sm/RNP and 3 for DNA), raising the possibility that the differences in IFN5 levels between groups are associated with differences in the autoantibody profile rather than the presence or type of clinical disease activity. Notably, the majority of the ANA^+^ individuals with detectable IFN-α levels had anti-Ro with or without anti-La antibodies (2 of 3 asymptomatic ANA^+^, 5 of 7 UCTD and 13 of 16 early SARD), with most of the remainder having a combination of anti-Sm/RNP with anti-RNA and anti-chromatin antibodies, with or without anti-Sm antibodies (2 of 7 UCTD and 2 of 16 early SARD).

### Associations between high IFN5 score and clinical symptoms in ANA^+^ individuals without SARD

To further explore the potential clinical significance of elevated levels of IFN-induced gene expression in the ANA^+^ non-SARD groups, the association between clinical symptoms and IFN5 levels was examined. For individuals who were ANA^+^ and lacked any clinical SARD classification criteria, we investigated whether there was an association between the clinical symptoms prompting ANA testing and the IFN signature. Of the 38 individuals in this group, 10 were found to be ANA^+^ after recruitment as HC, 4 after delivery of an infant with neonatal lupus or congenital heart block (all anti-Ro antibody–positive), 15 following investigation for arthralgia/myalgia, 6 following investigation for skin symptoms and 3 following investigation for other symptoms (1 eye symptoms, 1 white digits in the absence of Raynaud’s phenomenon and 1 headache). The frequency of elevated IFN5 scores varied in these groups, with 100% of Ro^+^ mothers, 40% of ANA^+^ HC, 33% of individuals with skin symptoms, 20% of individuals with arthralgia/myalgia and none of the individuals with other symptoms having high IFN5 levels. Amongst those individuals with elevated IFN5 scores, the highest levels were seen in Ro^+^ mothers (range 30,536–129,159). No difference was seen between the IFN5 levels in those individuals who were originally recruited as HC (range 26,074–71,028) and those who had non-specific symptoms (range 20,382–73,333).

Of the 28 patients with UCTD, 6 had symptoms suggestive of SSc (pre-SSc; Raynaud’s phenomenon, oesophageal dysmotility, telangiectasia and digital ulcers) in the absence of scleroderma/sclerodactyly, 2 had sicca symptoms and the remainder had symptoms suggestive of incomplete lupus syndrome (ILE; arthritis, lupus rashes, vasculitic skin lesions, pleuritis, idiopathic thrombocytopenic purpura, pericarditis and mucocutaneous ulcers). Notably, none of the patients with pre-SSc had high IFN5 levels, whereas 50% of patients with ILE and 100% of patients with sicca symptoms (both Ro^+^) had high IFN5 levels.

Although we plan to follow all patients without SARD on a yearly basis or earlier if new symptoms develop, we currently have limited follow-up data on these individuals because many were fairly recently recruited. One-year follow-up information is available for 19 of the ANA^+^ individuals who lacked clinical SARD criteria. Four patients have developed SARD symptoms, three of whom developed inflammatory arthritis (two were anti-Ro^+^, both with high IFN levels, one of whom had detectable IFN-α) and one of whom developed myositis (anti-RNP^+^ with a normal IFN5 level). Although there was a trend for an increased proportion of individuals with high (2 of 5) as compared with normal (2 of 14) IFN5 levels who developed SARD symptoms, this did not achieve statistical significance. Within the UCTD subset, 1-year follow-up information is available for 23 patients. Of these, seven have developed new SARD symptoms, six of whom have sufficient classification criteria for a diagnosis (three with SLE, one with SS, one with SSc and one with rheumatoid arthritis). The remaining patient, who was anti-Ro^+^ and who presented with a transient episode of inflammatory arthritis, developed new onset of sicca symptoms. Of the 12 patients with high IFN5 scores, 4 progressed (2 to SLE, 1 to SS and 1 with new sicca symptoms, and 3 of whom had high serum levels of IFN-α). This rate of progression was not significantly different from that of the 11 patients with normal IFN5 scores, among whom 3 progressed (1 each to rheumatoid arthritis, SSc and SLE). There was no association between age and the risk of progression in either of the ANA^+^ non-SARD groups, either as a whole or when the subset of individuals with high IFN5 scores was examined.

## Discussion

Type I IFNs have been proposed to play an important role in the pathogenesis of SARD through a potential feed-forward mechanism in which elevated levels of nuclear antigen–containing immune complexes lead to enhanced production of type I IFNs, which in turn further disturbs B- and T-cell tolerance mechanisms promoting production of ANAs [24]. Consequently, it has been hypothesized that elevated levels of type I IFNs may accompany and drive the conversion from pre-clinical to symptomatic autoimmunity in SARD [24]. In support of this concept, administration of type I IFNs to patients with hepatitis or malignancy leads to development of SARD in a small subset of patients, which abates once treatment is discontinued [25, 26]. In addition, the majority of patients with SARD have blood changes consistent with elevation of type I IFNs [11–16], and a number of SARD genetic risk variants have been shown to enhance the production of and/or the response to these IFNs [27]. Although there is some data suggesting that elevations of levels of serum IFN-α are present in a subset of healthy family members of patients with SLE and display features consistent with a heritable trait [28], it is not known whether elevation in type I IFNs precedes and/or predicts the conversion to symptomatic autoimmunity in ANA^+^ individuals. In this study, as a preliminary means of addressing this question, we asked whether increased levels of type I IFNs, as measured by an IFN signature, can be seen in ANA^+^ individuals who either lack or have insufficient criteria for a SARD diagnosis. We show that approximately one-third of ANA^+^ individuals lacking any clinical SARD criteria have elevated levels of IFN-induced gene expression. Although it could be argued that these individuals were not truly asymptomatic because they had clinical symptoms that led to their ANA testing, the same findings were observed for clinically asymptomatic Ro^+^ mothers and individuals who were recruited as HC but were subsequently found to be ANA^+^. Thus, the presence of an IFN signature clearly is not temporally associated with the presence of clinical SARD symptoms in a significant subset of ANA^+^ individuals. Notably, comparable elevations in IFN-induced gene expression were not seen in any of the ANA^−^ HC (only one of whom has an IFN5 score slightly above the normal range). Indeed, all HC who had high IFN signatures were subsequently found to have ANAs, a finding that suggests that the elevated levels of IFN-induced gene expression are closely associated with ANA production. To some extent, these findings recapitulate those seen in individuals with established SLE, in whom the presence of an IFN signature was more closely associated with autoantibodies than with clinical disease activity [22, 29].

Elevated levels of IFN-induced genes were also seen in a significant subset of patients with UCTD. Previous work has shown that about 50% of patients with ILE have elevated IFN signatures [30], and our findings are consistent with these observations. However, we have extended this work by examining not only patients with ILE but also other patients with pre-SARD. We show that patients with pre-SS have elevated IFN signatures, whereas these changes are not seen in patients with pre-SSc. Although a significant proportion of patients with SSc with established disease (47–68%) are reported to have elevated IFN-induced gene expression in their peripheral blood, the levels of expression are generally lower than those seen in SLE [11, 15]. In the present study, we show that only about 35% of patients with newly diagnosed SSc have an elevated IFN signature in their whole peripheral blood, with lower mean levels in these patients than in other early SARD groups. This finding, taken together with the lack of IFN signature elevation in patients with pre-SSc, one of whom subsequently developed SSc, suggests that elevations in IFN may occur later in the disease course of SSc and/or may play a less important pathogenic role than in SLE and SS. However, given the relatively small number of patients with SSc examined, this finding requires validation in an independent cohort of patients with newly diagnosed SSc.

In ANA^+^ individuals without SARD, elevated levels of IFN-induced gene expression correlated with serologic findings. Although previous researchers examining patients with established SARD have shown that elevations in anti-chromatin, anti-dsDNA, anti-RNP, anti-Ro, anti-La and anti-Scl70 antibodies are associated with elevated levels of IFN-induced gene expression [11, 14–16, 22, 24, 31, 32], amongst the asymptomatic ANA^+^ individuals with detectable specific anti-nuclear autoantibodies, only anti-Ro and/or anti-La antibodies were associated with an elevated IFN signature. Indeed, of the seven individuals with these autoantibodies, only one did not have a high IFN5 level. In addition, one HC who was ANA^−^ and therefore did not satisfy our inclusion criteria was anti-Ro^+^ and had a high IFN5 level (67,233). Thus, anti-Ro antibodies are closely associated with type I IFN expression even in HC.

However, not all anti-Ro^+^ individuals had high levels of IFN-induced gene expression, including a subset of those with UCTD or early SARD, indicating that other factors are also required to lead to the generation of IFN in these individuals. Previous work has shown that production of IFN in anti-Ro^+^ individuals is associated with the lupus risk variant of *IRF5* [24, 33], and thus these individuals may lack genetic variants that promote IFN production. Alternatively, these individuals may lack a source of the nuclear autoantigens that are presumably required to produce the immune complexes that drive IFN production by plasmacytoid dendritic cells. It is currently unclear what processes lead to the production of these immune complexes in asymptomatic ANA^+^ individuals.

Our findings contrast with the results of a previous study that suggested that IFN levels are not elevated in asymptomatic Ro^+^ individuals [34]. In that study, the ability of serum from anti-Ro^+^ mothers of infants with neonatal lupus to promote IFN-induced gene expression in an indicator cell line was examined (serum IFN activity). Serum IFN activity levels in 24 asymptomatic mothers were found to be similar to those of healthy control subjects, whereas 25–75% of mothers with pre-SLE, SLE, pre-SS or SS had elevated serum IFN activity. The origin of the differences between the two studies likely lies in the different techniques used to measure IFN, with serum IFN activity reflecting the amount of type I IFN in the serum, whereas an IFN signature measures exposure of peripheral blood cells to type I IFN, which could occur in either the tissues or blood. In keeping with previous work indicating a modest correlation between serum IFN activity and the IFN signature in SLE [35], we found a moderate correlation between serum IFN-α levels and IFN-induced gene expression as measured by the IFN5 score in all ANA^+^ groups. Notably, increased serum IFN-α levels were detected only in ANA^+^ individuals with very high IFN5 scores and demonstrated considerable variability between samples with similar scores in this range. It is currently unclear whether this variability represents differences in the site of, and/or the immunologic mechanism leading to, IFN production. Nevertheless, in contrast to the previous study [34], we found that IFN-induced gene expression, and in some cases detectably elevated levels of serum IFN-α, could be just as high in asymptomatic anti-Ro^+^ individuals as in patients with early SARD, suggesting that the amount of IFN to which their peripheral blood cells are exposed is similar.

Our results are in agreement with those reported in a recent publication on the temporal relationship between cytokine elevation, autoantibodies and the development of lupus classification criteria in a longitudinally followed cohort prior to the development of SLE. As observed in that study, we also found that immunologic changes indicating elevated levels of type I IFN were not seen in the absence of ANAs and correlated with the number of autoantibody specificities that were present [36]. However, in contrast to that study, we saw elevated IFN5 scores in individuals who lacked specific autoantibodies as detected by the BioPlex® 2200 ANA screening system. One reason for this apparent discordance may be that the majority of our study participants were Caucasian, whereas those in the study by Munroe et al. study [36] were predominantly of African or other non-Caucasian descent. Previous work has shown that the BioPlex® 2200 ANA screening system is significantly less able to detect ANAs in Caucasians than the ANA detected by immunofluorescence, whereas the agreement between these two assays is much better for other ethnic groups [37]. Nevertheless, our results provide further support for the concept that elaboration of type I IFN occurs as a consequence of autoantibody production rather than as an initial etiologic mechanism that leads to their generation.

Despite the short duration of follow-up for the individuals without SARD in our study, approximately 20% of asymptomatic ANA^+^ individuals developed SARD symptoms/signs and 30% of UCTD patients evolved, with the majority meeting classification criteria for a SARD. Although there was an insufficient number of progressing individuals to determine whether progression to any SARD or a specific SARD might occur more frequently in individuals with elevated levels of IFN-induced gene expression, it is clear that an increased IFN5 score is not strongly associated with imminent progression over the next year to SARD. Conversely, progression to a SARD classification occurred in some individuals in the absence of elevated IFN-induced gene expression (confirming and extending the findings of Munroe et al. for SLE [36]).

## Conclusions

An IFN signature that is quantitatively and qualitatively similar to that seen in SARD is found in a significant proportion of ANA^+^ individuals who lack or have insufficient clinical classification criteria for a diagnosis of SARD. In these individuals, elevated levels of IFN-induced gene expression correlated with ANA titre and the presence of anti-Ro antibodies, but they were not required for or predictive of clinical progression over the subsequent year. Thus, it is unlikely that an elevated IFN signature will be the sole predictive factor for SARD disease progression, and additional novel biomarkers, or algorithms using existing biomarkers, must be sought.
